# Sero-prevalence and associated risk factors of Brucellosis among Malaria negative febrile out-patients in Wakiso district, Central Uganda

**DOI:** 10.1186/s13104-018-3907-3

**Published:** 2018-11-08

**Authors:** Samuel Majalija, Patrick Luyombo, Gabriel Tumwine

**Affiliations:** 1 Department of Biosecurity, Ecosystem and Veterinary Public Health, College of Veterinary Medicine, Animal Resources and Biosecurity, Makerere University, P.O. Box 7062, Kampala, Uganda; 2 Department of Biomolecular Resources and Biolab Sciences, College of Veterinary Medicine, Animal Resources and Biosecurity, Makerere University, P.O. Box 7062, Kampala, Uganda; 3 Namayumba Health Centre IV, Wakiso District, Uganda

**Keywords:** Brucellosis, *Brucella abortus*, Public health, Raw milk, Zoonoses

## Abstract

**Objective:**

Brucellosis is a zoonotic disease usually acquired through direct contact with the infected animals and consumption of contaminated milk and meat products. In humans Brucellosis presents similar signs with other febrile diseases like Malaria, typhoid and other febrile conditions. This study was carried out to determine the prevalence of *Brucella abortus* among patients with fever but were negative for Malaria.

**Results:**

A cross-sectional study was carried out in Namayumba Health Centre IV, Wakiso district involving 200 participants. Blood samples was screened for *B. abortus* using Serum Agglutination Test and confirmed with Tube Agglutination test. A questionnaire was used to collect data on socio-demographic characteristics and human Brucellosis related risk factors. Human *B. abortus* sero-prevalence was at 7.5% (n = 200). The prevalence was high among participants aged 18–35 years (13.3%), muslims 12 (14.0%), those with no formal education (33.3%) and divorced 2 (14.3%). Consuming of raw milk (OR 2.162, 95% CI 0.021–1.379) and being a Muslim (OR 6.101, 95% CI 1.601–23.248) were associated with increased risk of *Brucella abortus*. It was concluded that human Brucella infection due to *Brucella abortus* is commonly associated with consumers of raw milk products and muslims in Wakiso district.

## Introduction

Human Brucellosis is a major zoonotic disease of global health importance causing for more than 500,000 new infections every year [1]. In humans the disease is associated with chronic debilitating infections, recurring febrile conditions, joint pains, malaise, fatigue and arthritis resulting in substantial residual disability [2]. Clinical signs in livestock include reduced fertility and abortions which cause severe economic losses to the farmers [3]. The disease is among the top neglected zoonoses that cause severe social economic impact in low income countries like Uganda. Largely human Brucellosis is caused by two species of *B. abortus* and *B. melitensis* [4], acquired through contact with infected animals, their tissues and products [2]. It is an occupational hazard to persons engaged in high risk professions especially the veterinarians; laboratory, slaughterhouse workers, and farmers [5, 6]. Other risk factors are associated with Brucellosis, include consumption of raw or unpasteurized milk and dairy products processed from such milk [7, 8].

The lack of sufficient data is the reason as to why the true incidence of Brucellosis remains unclear in low and middle-income Brucellosis-endemic countries, Nonetheless, previous reports indicate in the Middle East, Mediterranean region, Northern and Sub-Saharan Africa; human Brucellosis prevalence range from 5 to 55%; and 8 to 46% in animals [3, 9–11]. In Uganda a prevalence of 7–42.2% among cattle and goats has been reported [12], posing a big threat to abattoir workers and consumers. Human Brucellosis sero-prevalence was at 17% among agro-pastoral communities in Kiboga district, Central Uganda and therefore an important public health problem [8].

Equally, Malaria is an endemic disease in Uganda with prevalence as high as 70% in patients with pyrexia of unknown origin [13]. In a previous study, 73% of patients with joint pain, general malaise, and constant headache were infected with Malaria and 13.3% had Brucellosis [6]. Of greatest concern, however, is the lack of distinguishing clinical features between Malaria and human Brucellosis which confounds diagnosis in absence of laboratory confirmation [14]. Routinely patients with febrile conditions are screened and treated for Malaria as recommended [15], on the other hand, patients with Brucellosis are prone to misdiagnosis and erroneous treatment for Malaria. Such individuals suffer from protracted ill-health and fail to conduct their daily activities, through being unwell [7, 16]. Several studies focusing on prevalence of human Brucellosis in Africa and Uganda in general have been reported [3, 8–11], however, there is scanty information on the prevalence of Brucellosis among the febrile non-Malaria cases. This study therefore determined the sero-prevalence of Brucellosis as well as risk factors associated with occurrence of the disease among febrile non-Malaria out-patients attending Namayumba health centre IV in Uganda.

## Main text

### Methods

#### Study design, site and population

A cross sectional study was carried out among 200 out-patients at Namayumba Health Centre IV and presenting with fever at the time of seeking medical care between January and March 2016. The study excluded infants, patients without fever, and confirmed febrile Malaria positive patients. The sample size was determined as previously described [17], at 95% confidence interval (CI) and presumed sero-prevalence of 17% [8]. The strategic location of Namayumba Health Centre IV along the highway enables the communities from 15 villages of Wakiso district ease access to the health facility.

To establish the associated risk factors for Brucellosis information on patient identification, socio-demographic data, economic activity, feeding habits and knowledge was obtain using a questionnaire survey.

A venipuncture on the brachial vein was used to obtain 3–5 ml of blood collected into plain sterile vacutainer and allowed to clot at room temperature. Serum was harvested by centrifugation at 3000 rpm for 5 min and stored in serum vials and kept at 4 °C. Later, vials were transported on ice to the central diagnostic laboratory at the College of Veterinary Medicine, Animal Resources and Biosecurity, Makerere University for further processing. In the laboratory, Rose Bengal Plate Test (RBPT) and Serum Agglutination Test (SAT) [18] were carried out using *B. abortus* antigens (Veterinary Laboratory Agency, VLA, Weybridge, United Kingdom) to detect the Brucella antibodies. Only samples that gave signals for both RBPT and SAT with a titer of 1:160 or greater indicated sero-positivity were considered positive as recommended by OIE and other reports [19].

#### Data management and analysis

Data collected was recorded in the Laboratory record book, later entered into excel and transferred to the Statistical Package for Social Scientists (SPSS) software version 18 for analysis. Prevalence was obtained by descriptive analysis where frequencies and proportions were obtained. Risk factors were analyzed at univariate and bivariate level, using Chi square and p values < 0.005 were taken as statistically significant. Those that turn out to be statistically significant were further subjected to multivariate analysis to eliminate confounders at 95% confidence interval (CI).

### Results

#### Demographic characteristics of the study population

Table 1 presents descriptive characteristics of the participants. A total of 200 human participants were involved in the study. The age ranged from 5 to 62 years with overall average age of 44.7 (± 13.0) years with the majority 41.5% (n = 83) in 18–35 age group. A majority were female 55.5% (n = 111), employed 34.0% (n = 68), had primary education 51.5% (n = 103); 57.0% (n = 114) were Christians and more than a half of the participants were married 61.5% (n = 123).

**Table 1:** Descriptive socio-demographic characteristics of *B. abortus* among participants

Characteristics	Frequency (n)	% participants (95% CI) N = 200
Sex		
Male	89	44.5 (38.0–51.5)
Female	111	55.5 (48.5–62.0
Age (years)		
< 17	46	23.0 (17.5–29.0)
18–35	83	41.5 (34.5–48.0)
> 35	71	35.5 (29.0–42.5)
Religion		
Christian	114	57.0 (50.5–63.5)
Muslim	86	43.0 (36.5–49.5)
Occupation		
Employed	68	34.0 (27.5–41.0)
Unemployed	65	32.5 (26.0–39.5)
Self employed	60	30.0 (24.0–36.5)
Others	7	3.5 (1.0–6.0)
Education level		
None	3	1.5 (0.0–3.5)
Primary	103	51.5 (44.5–58.5)
Secondary	78	39.0 (32.0–46.0)
Tertiary	16	8.0 (4.5–12.0)
Marital status		
Married	123	61.5 (55.0–68.0)
Single	63	31.5 (25.0–38.0)
Divorced	14	7.0 (3.5–11.0)

#### The sero-prevalence of Brucellosis in the study population

The overall sero-prevalence of Brucellosis among the 200 participants was 7.5% (n = 15) as shown in Table 2. It was highest among participants aged 18–35 years (13.3%, n = 11), varied among males (7.9%, n = 7) and females (7.2%, n = 8). The prevalence was also higher among Muslim (14.0%, n = 12), divorced (14.3%, n = 2), and among employed (10.3%, n = 7) participants. Most of the social-demographic factors were not associated (p > 0.05) with the occurrence of *B. abortus* except age (p = 0.031) and religion (p = 0.003) (Table 2).

**Table 2:** Prevalence of *B. abortus* among participants

Characteristics	Frequency	+ve *B. abortus* (n, %)	p-value
Sex			
Male	7	7.9 (3.4–14.6)	0.861
Female	8	7.2 (2.7–11.7)	
Age (years)			
< 17	1	2.2 (0.0–6.5)	0.031
18–35	11	13.3 (6.0–21.7)	
> 35	3	4.2 (0.0–8.5)	
Religion			
Christian	3	2.6 (0.0–6.1)	0.003
Muslim	12	14.0 (7.0–22.1)	
Occupation			
Employed	7	10.3 (2.9–17.6)	0.295
Unemployed	2	3.1 (0.0–7.7)	
Self employed	6	10.0 (3.3–18.3)	
Others	0	0.0 (0.00–0.00)	
Education level			
None	1	33.3 (0.00–100)	0.382
Primary	8	7.8 (2.9–13.6)	
Secondary	5	6.4 (1.3–12.8)	
Tertiary	1	6.3 (0.0–18.8)	
Marital status			
Married	12	9.8 (4.1–15.4)	0.082
Single	1	1.6 (0.0–4.8)	
Divorced	2	14.3 (0.0–35.7)	

#### Risk factors for Brucellosis in the study population

Aside from the consumption of raw milk which was significantly associated with Brucellosis infection (p = 0.038), the rest of the potential risk factors did not have any significant influence (Table 3).

**Table 3:** Potential risk factors for the occurrence of *B. abortus* among humans

Attribute	Frequency (n)	% participants N = 200	Positive *B. abortus* (n, %)	Unadjusted OR (95% CI)	p Value
Have animals at home					
No	101	50.5	5 (5.0)	2.157 (0.710, 6.556)	0.167
Yes	99	49.5	10 (10.1)		
Knowledge on Brucella					
No	140	70.0	14 (10.0)	0.153 (0.020, 1.188)	0.064
Yes	60	30.0	1 (1.7)		
Consume raw milk					
No	172	86.0	2 (1.2)	0.921 (0.884, 0.960)	0.038
Yes	28	14.0	13 (46.4)		
Consume milk products					
No	163	81.5	10 (6.1)	2.391 (0.765, 7.468)	0.124
Yes	37	18.5	5 (13.5)		
Share water source with animals					
No	174	87.0	15 (8.6)	0.914 (0.873, 0.956)	0.120
Yes	28	13.0	0 (0.0)		
Deal with animal products					
No	107	53.5	6 (5.6)	1.804 (0.617, 5.273)	0.276
Yes	93	46.5	9 (9.7)		

Both socio-demographic and potential risk factors that showed p-values < 0.05 in the bivariate analysis were considered and included in the final multivariable regression model. Consumption of raw milk (OR 2.162, 95% CI 0.021–1.379) and being a Muslim (OR 6.101, 95% CI 1.601–23.248), increased the odds of being infected with *B. abortus* among participants.

### Discussion

Brucellosis occurs naturally in animals, while humans get infected through contact with the infected animal and consumption of contaminated animal products [2, 20]. Therefore, it has been shown that prevalence of human Brucellosis tends to correspond to that in animals [21, 22].

In the present study, *B. abortus* sero-prevalence was 7.5% which is in congruence with 8% human prevalence of Brucellosis reported in Egypt and 10% among abattoir worker in Uganda [12]. However, the overall prevalence is expected to be higher than was observed because only febrile non- Malaria cases were tested for Brucellosis. Individual cases with Malaria plus Brucellosis were no included in the study, whereas, previous studies have reported Malaria-Brucellosis co-infections in febrile cases [23]. In concurrence, with this observation, earlier studies reported much higher prevalence of 21.2% among patients with febrile clinical signs and 44% prevalence was reported among butcher workers in northern Nigeria [24, 25].

In our study, consumption of raw milk, and being a Muslim were risk factors associated with *B. abortus*. Evidently, consumers of raw or unpasteurized milk were two times more likely to test positive for *B. abortus* than those who don’t. This is in not surprising since Brucella positive cows shed the organisms in milk which is a major vehicle for human Brucella infection [20, 26, 27]. This finding also strengthens the argument that humans get infected through consumption of contaminated animal products as earlier reported [5, 27]. Therefore, efforts to reduce human Brucella infection should target provision of pasteurized milk or milk boiling centres from where the communities can access treated safe milk. The provision milk boiling centres has been suggested as an affordable option that would greatly reduce risk of Brucellosis without affecting livelihoods of the poor people associated with the milk production and dairy value chain in Uganda [21].

The study also observed that being a Muslim was associated with occurrence of *B. abortus*, to the extent that Muslims were six times more likely to test positive for *B. abortus* than Christians. This could be explained by the fact that slaughter of animals with exception of pigs is entirely carried out by muslins in Uganda. These constitute a majority of livestock traders, butchers and others working closely with cattle for slaughter which increases exposure and infection with Brucella organisms. Clearly the religion of muslins has significant influence on the occupational risks and exposure to infected animals with Brucellosis. Our results are supported by other studies that have shown that transmission of Brucellosis can occur through consumption of raw or undercooked meat, blood or offal consumption [5, 28]. These further support the arguments that religion among other factor significantly influence the spread and control of zoonotic diseases in Africa [29, 30].

There was no statistically significant correlation between occurrence of *B. abortus* and having animals at home, knowledge on Brucella, consuming milk product, sharing water source with animal and dealing with animals’ products. These findings are in contrast to earlier studies that reported that Brucellosis occurrence in humans was associated with having animals at home [31], consuming milk products [32, 33], sharing water source with animals [34] and dealing with animal products [6, 12]. The variation in the risk factors between Uganda and other countries is likely to be associated with the diverse socio-cultural and anthropological attributes among the various countries.

### Conclusion

*Brucella abortus* infection among people living in Busiro North, Wakiso district was at 7.5% which could be a public health concern if not controlled at this moderate prevalence. This study revealed consumption of raw milk and being a Muslim as risk factors associated with *B. abortus* infection in humans. Public awareness campaigns target consumption of treated milk and personnel that handle and slaughter animals in abattoirs to reduce spread of *B. abortus*.

## Limitations of the study

Aside from Brucellosis, other bacterial causes of septicemia leading to fever were not considered which is a shortcoming of our study. This was an oversight which would have given important information on the febrile cases of unknown origin that were negative for Malaria and Brucellosis.

## Abbreviations

RBPT: Rose Bengal Plate Test
SAT: Serum Agglutination Test
SPSS: Statistical Package for Social Scientists
